# Construction and characterization of avermectin B_2_ solid nanodispersion

**DOI:** 10.1038/s41598-020-66098-3

**Published:** 2020-06-04

**Authors:** Bo Cui, Fei Gao, Zhanghua Zeng, Chunxin Wang, Yan Wang, Changjiao Sun, Xiang Zhao, Liang Guo, Yue Shen, Guoqiang Liu, Haixin Cui

**Affiliations:** Institute of Environment and Sustainable Development in Agriculture, Chinese Academy of Agricultural Sciences, Beijing, 100081 China

**Keywords:** Engineering, Materials science, Nanoscience and technology

## Abstract

Poorly water-soluble pesticide compounds are difficult to be formulated as environmentally friendly formulations with high efficacy. For the conventional formulations, more than 50% of pesticides are lost during application due to the decomposition of active ingredient, dust drift and running off. Therefore, there is an urgent need to construct a novel formulation for improving the bioavailability of pesticides. The avermectin B_2_ solid nanodispersion was developed by self-emulsifying and solidification technology. The average particle size, surface tension and contact angle on cabbage leaves of the solid nanodispersion were 35.3 nm, 36.6 mN/m and 58°, respectively. The toxicities of the nanoformulation against diamondback moths and root-knot nematode were more than 1.7 times that of conventional emulsion in water and water dispersible granule. This investigation demonstrated that for foliage-applied pesticides, the formulation bioavailability had positive correlation with wettability which was negatively correlated with surface tension and contact angle. This study provides an easy and scalable technique to construct the effective and environmentally friendly nanoformulations. The toxicity improvement of the solid nanodispersion will significantly reduce dosage and environmental pollution of pesticide. The clarified relationship between formulation parameters and biological activity will contribute to the design and construction of novel pesticide formulations.

## Introduction

Pesticide is a kind of primary agrochemical to control weeds, pests and plant diseases for ensuring and improving crop yields. However, most pesticide compounds are poorly soluble in water which limits the development of their formulations with high efficacy and safety. For the conventional pesticide formulations, more than 50% of pesticides have been lost during foliar spraying caused by the decomposition of active ingredient, dust drift and running off^1,2^. The actual utilization of target organisms is less than 0.1%^3,4^. In addition, a large amount of organic solvents or emulsifiers are used to maintain the formulation stability and effectiveness in emulsifiable concentrate (EC) and microemulsion. The low efficacy and environmentally unfriendly composition of conventional formulations would lead to resource waste and a series of food safety and environmental pollution issues^5,6^. In this situation, it has great significance in investigating the relationship between the formulation characteristics, especially the effect of formulation parameters on bioavailability. It can provide a theoretical basis for the design and construction of highly effective and safe formulations.

Avermectins are a family of 16-membered macrocyclic lactones isolated from *Streptomyces avermitilis*^7^. The family consists of eight components with similar chemical structures (designated A_1a_, A_1b_, B_1a_, B_1b_, A_2a_, A_2b_, B_2a_, B_2b_). Though all the avermectins have nematocidal, acaricidal and insecticidal activity combined with low toxicity to mammals, the B-series components have been proven to be more active than the A-series^7^. At present, the main product in the market is avermectin B_1_ which has optimum efficacy against mites and insects. However, in recent years, there has been growing attention on avermectin B_2_ because it is more effective against root-knot nematode^8–10^. The poor water solubility of avermectin B_2_ reduces its dispersibility in water, and further affects the shelf life and bioavailability of products. Therefore, the main formulation of avermectin B_2_ was still EC^10,11^. It is necessary to develop a simple method to prepare high-efficiency and environmentally friendly formulations for avermectin B_2_ as well as other poorly soluble pesticides.

Nanotechnology provides a promising approach to improve formulation performance by nanosizing. According to the Ostwald-Freundlich and Noyes-Whitney equations, the saturation solubility and dissolution rate increase with decreasing particle size^12,13^. Therefore, the small size and large specific surface area of nanoparticles are beneficial to improve the dispersibility of water-insoluble pesticides, and further enhance their spreading, coverage and retention on leaves and target organisms^14,15^. The techniques for producing nanoformulations involve top-down and bottom-up methods. The top-down technique prepares nanoparticles by breaking down bulk materials or larger particles into small ones^16^. Wet milling and high-pressure homogenization are typical top-down methods. Although they are less complicated, the in-process heat generation and need for high-cost equipments are their disadvantages. The bottom-up approaches like microprecipitation and supercritical fluid build nanoparticles by assembling molecules or smaller particles together^17,18^. However, the procedure parameters of the bottom-up process usually need to be precisely controlled. Self-emulsifying is a major technology for preparing microemulsion and nanoemulsion^19,20^. Hydrophobic active ingredients are wrapped into amphiphilic micelles and stably suspended in solution. This technique is simple, energy-saving and easy to scale up. Nevertheless, the relevant reports on solid nanoformulation produced by self-emulsifying are rare.

In order to improve pesticide bioavailability and overcome the defects of conventional formulations, the present research focuses on two objectives. One is to build a nanoformulation for enhancing the biological activity of pesticides through nano-size effect. The other is to reveal the relationship between formulation parameters and biological activity for guiding the design and construction of novel formulations. Meanwhile, avermectin B_2_ as a potentially effective nematocide has not been widely used, so it is an ideal research object for poorly soluble pesticide compounds. Based on the above, this investigation proposed a self-emulsifying combined with solidification method to construct the avermectin B_2_ solid nanodispersion. The particle size, morphology, interfacial charge and stability of the solid nanodispersion have been characterized. The surface tension, contact angle and bioavailability of different formulations have been compared and the relationship between the formulation characteristics has been further investigated. The highly effective solid nanodispersion prepared by the simple process could significantly reduce the pesticide dosage and frequency of administration compared to the conventional formulations, thus improving its environmental and ecological security.

## Results and Discussion

### Carrier screening

Based on our previous research^21^, the composite surfactant of emulsifier 600 and 700 could effectively stabilize avermectin class nanoparticles in the self-emulsifying system, so this surfactant combination was also selected to produce the avermectin B_2_ solid nanodispersion. In the formulation composition, water-soluble carrier can not only adsorb pesticide solution but also accelerate redispersion of the solid powder. It has an important influence on the particle size, dispersibility and suspensibility of the formulation, so the carrier was optimized in this investigation. The 4.0% (w/w) avermectin B_2_ solid nanodispersions with different kinds of carriers were prepared. As shown in Table 1, the mean size of the nanoparticles using sodium benzoate as carrier was 43.9 nm, smallest among the four formulations. In contrast, the particles of the other three systems were larger than 150 nm. As an evaluation index of the size distribution, polydispersity index (PDI) less than 0.3 is conducive to improving the system stability. In this research, only the sodium benzoate solidified nanoparticles presented the narrow size distribution, implying superior dispersibility and uniformity. Considering the particle size and dispersion effect, sodium benzoate was a suitable carrier to prepare the avermectin B_2_ solid nanodispersion.

**Table 1:** Effect of carrier on the particle size and dispersibility of the avermectin B_2_ solid nanodispersions. S.D.: standard deviation of three measurements. Different letters at each data indicate significant differences according to Duncan’s multiple range test at P < 0.05.

Carrier	Mean size (nm) ± S.D.	PDI ± S.D.
Sodium benzoate	43.9 ± 0.5 c	0.244 ± 0.015 c
Glucose	159.2 ± 0.9 b	0.311 ± 0.015 c
Sucrose	235.5 ± 18.5 a	0.433 ± 0.024 b
α-Lactose	217.8 ± 6.1 a	0.537 ± 0.083 a

### Pesticide content optimization

**Figure 1:**
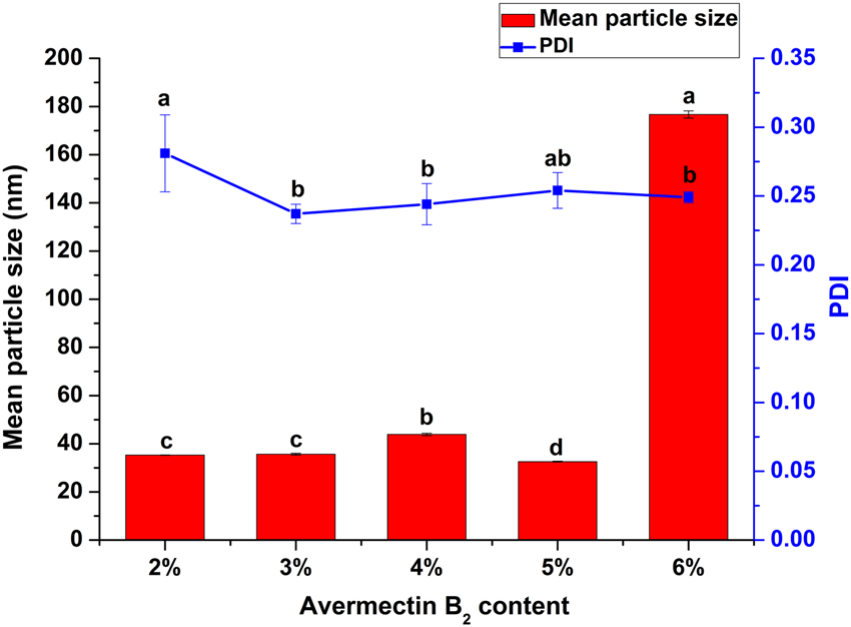
Particle sizes and dispersibilities of the solid nanodispersions with different avermectin B_2_ contents. Different letters at each data indicate significant differences according to Duncan’s multiple range test at P < 0.05.

High content of formulations are beneficial to reduce the costs of processing and application. However, the formulation content is limited by the solubility of active ingredient in solvent and the adsorption capacity of carrier. The solid nanodispersions containing 2% to 6% (w/w) avermectin B_2_ were prepared. As shown in Fig. 1, when the pesticide loading was in the range of 2% to 5% (w/w), the mean particle sizes were between 30 nm and 45 nm. As the avermectin B_2_ content rose to 6%, the active ingredient was easier to aggregate during carrier adsorption, so the particle size significantly increased to 176.7 nm. Therefore, in terms of particle size and distribution, the suitable pesticide content was 2% to 5% (w/w). However, as the pesticide content increased, the amount of ethyl acetate in the preparation process also gradually increased in order to dissolve the active ingredient adequately, thus resulting in a longer drying time. As a consequence, considering energy consumption, cost and practical application, the 2% (w/w) content was preferable. In the following, the 2% (w/w) avermectin B_2_ solid nanodispersion was characterized in detail.

### Size and morphology of the nanoparticles

**Figure 2:**
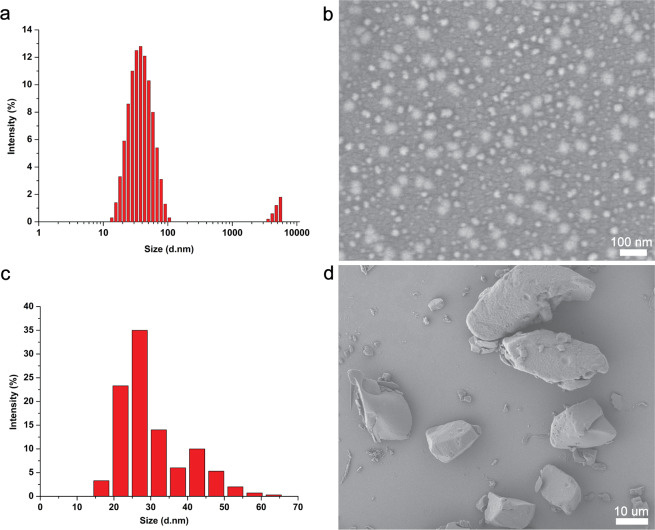
The sizes and morphologies of the avermectin B_2_ nanoparticles and TC. (**a**) size of the nanoparticles measured by DLS; (**b**) SEM image of the nanoparticles with magnification of 60000; (**c**) statistical particle size based on SEM image; (**d**) SEM image of TC with magnification of 1000. Size (d. nm): diameter of the nanoparticles.

Compared with EC and emulsion in water (EW), the formulation composition with 2.0% (w/w) avermectin B_2_, 4.0% (w/w) emulsifier 600, 4.0% (w/w) emulsifier 700 and 90.0% (w/w) sodium benzoate is free of organic solvent, so it is safe and environmentally friendly. The mean particle size and PDI of the solid nanodispersion measured by dynamic light scattering (DLS) were 35.3 ± 0.1 nm and 0.281 ± 0.028 nm, respectively (Fig. 2a). As observed from the SEM image (Fig. 2b), the avermectin B_2_ nanoparticles were mainly spherical or ellipsoidal in shape. The particle size counted from 300 particles in the SEM image was between 14.9 nm and 65.6 nm, with an average diameter of 30 nm (Fig. 2c). The statistical value was consistent with the DLS result. By contrast, the particles of the avermectin B_2_ technical material (TC) presented an irregular blocky structure with a micron size (Fig. 2d) because of its poor solubility and dispersibility in water. The small size and uniformity of the solid nanodispersion could help improve the redispersibility and stability of the formulation.

### Zeta potential and pH

Aqueous dispersions with absolute values of zeta potential higher than 30 mV are generally considered to have long-term stability^22^. However, when the surfactants could provide both steric hindrance and electrostatic repulsion, the shear plane may shift to a larger distance from the particle surface, which further leads to a decrease in zeta potential^22^. In this investigation, the zeta potential of the re-dispersed nanodispersion was −4.1 ± 1.2 mV. This low potential value was attributed to the formulation composition, especially the type of surfactants. Both emulsifier 600 and 700 were nonionic polymers, so they could not provide strong charge when they adsorbed on the pesticide surface. However, the long hydrophobic chains of the two polymers could induce steric hindrance against aggregation. The steric effect and electrostatic repulsion synergistically maintained the system stability. In addition, the avermectin B_2_ solid nanodispersion was in solid state before application. A short-term stability after dilution with water could meet the spraying requirement. In contrast, the storage stability of the solid powder was more important. The pH of the re-dispersed nanodispersion was measured to be 7.3 ± 0.1. The neutral condition could avoid the decomposition of the sensitive active ingredient.

### Storage stability

**Figure 3:**
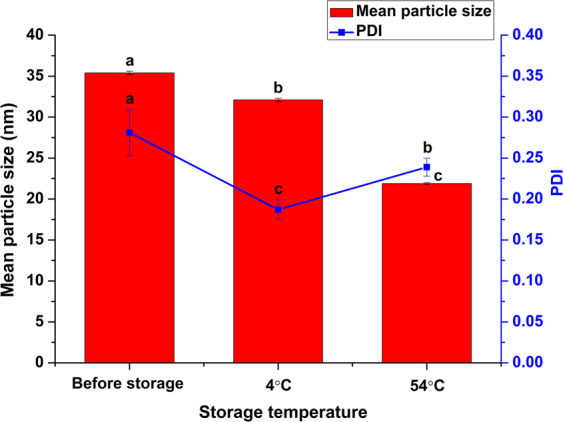
Storage stability of the avermectin B_2_ solid nanodispersion. Different letters at each data indicate significant differences according to Duncan’s multiple range test at P < 0.05.

The physical stability of the solid nanodispersion was evaluated according to CIPAC MT 46 and GB/T 19136–2003. As shown in Fig. 3, the mean size of the nanoparticles changed slightly after storage at 4 °C for 7 days. When stored at 54 °C for 14 days, the particle size reduced to 21.9 nm. Meanwhile, the PDI decreased to 0.239. This result indicated the solid nanodispersion had excellent storage stability. In the composition of the avermectin B_2_ solid nanodispersion, emulsifier 600 and 700 are liquid at room temperature. During storage at 54 °C, heating and the physical state of the surfactants were conducive to accelerating molecular motion and collision which may cause particle recombination. The decreased PDI proved that the particle size became more uniform. The similar phenomenon has been reported in other nanoformulations^23,24^. In addition, particle size reduction during storage has also been observed by other researchers^25^. However, the relevant mechanism is still unclear. One possible explanation is that the number of large particles decreased during particle reorganization at high temperature. Dynamic light scattering measures the agglomeration state of nanoparticles in suspension. Larger particles will dominate the light scattering signal and mask the presence of the smaller particles during DLS measurement^26^. Therefore, after storage at 54 °C, the system with fewer large particles and narrow size distribution exhibited smaller particle size. It is worth noting that in the 2.0% (w/w) avermectin B_2_ solid nanodispersion, the content of sodium benzoate as carrier accounted for 90.0% (w/w), so the physicochemical properties of sodium benzoate played a crucial role in maintaining the formulation stability. According to the literature^27^, crystals are more stable than amorphous particles because the amorphous state tends to recrystallize. The crystalline structure and high melting point of sodium benzoate can keep it stable during storage, thereby improving the stability of the solid nanodispersion.

### Surface tension

**Figure 4:**
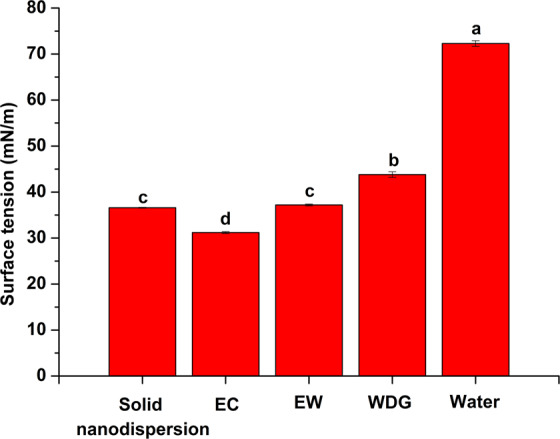
The surface tensions of the avermectin B_2_ formulations. Different letters at each data indicate significant differences according to Duncan’s multiple range test at P < 0.05.

For foliage-applied pesticides, the spreading and adsorption properties of droplets on the crop leaves are key factors affecting the effective utilization of active ingredient. Reduced surface tension can enhance wettability and further promote the deposition and retention of pesticide droplets on target surfaces^28^. Figure 4 shows the surface tensions of the avermectin B_2_ solid nanodispersion, EC, EW and water dispersible granule (WDG). The surface tensions of all the formulations were less than 45 mN/m. This value was much lower than the 72.3 mN/m of pure water because the surfactants or adjuvants in the formulation composition have a significant effect on surface tension reduction. Among the four kinds of formulations, the trend of surface tension was EC < solid nanodispersion <EW < WDG. This tendency was similar to the result reported in the previous literature in which the surface tension was EC < suspension concentrate <WDG^29^. Among these formulations, EC and EW usually contain organic solvents which can dramatically reduce surface tension. By contrast, the solid nanodispersion did not contain any solvent, the decrease in surface tension may be attributed to the formulation composition and particle size reduction.

### Contact angle

**Figure 5:**
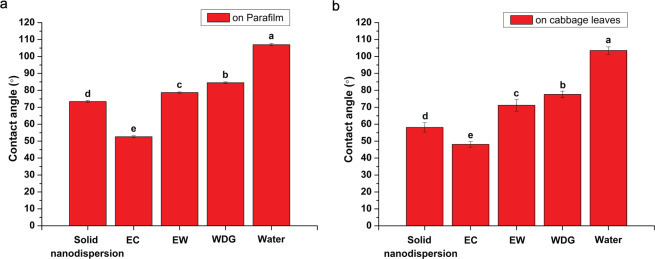
The contact angles of the avermectin B_2_ formulations on (**a**) Parafilm and (**b**) cabbage leaves. Different letters at each data indicate significant differences according to Duncan’s multiple range test at P < 0.05.

The contact angle of pesticide droplets is another typical indicator of formulation wettability. Many kinds of crop leaves are hydrophobic such as rice, wheat and cabbage. They are more difficult to be wetted than hydrophilic leaves, so it is more necessary to improve the pesticide wettability on hydrophobic crops. In this investigation, Parafilm and cabbage leaves were used as hydrophobic model substrates to evaluate the formulation wettability. As shown in Fig. 5a, the measurement results on Parafilm had better reproducibility and smaller standard deviation because its surface structure was more uniform. The contact angle of pure water on Parafilm was 107 ± 1°, which was in agreement with the literature^30^. In contrast, the contact angles of the avermectin B_2_ solid nanodispersion, EC, EW and WDG on Parafilm were 73°, 53°, 79° and 85°, respectively. As shown in Fig. 5b, the contact angles of the solid nanodispersion, EC, EW, WDG and water on cabbage leaves were 58°, 48°, 71°, 78° and 104°, respectively. The smaller contact angle indicated the better wettability. Regardless of the surface of Parafilm or cabbage leaves, the contact angles of all the formulations were less than 90° and much smaller than that of pure water, implying an increase in wettability on hydrophobic surfaces. Droplet behaviors varied with the fine structures of substrate surfaces, so the contact angles on different surfaces had a certain difference. However, the wetting capabilities of the four formulations on the two hydrophobic substrates were the same, EC > solid nanodispersion > EW > WDG. It is noteworthy that the contact angle presented the same trend as the surface tension, negatively correlated with the wettability. The formulation composition, especially the surfactant acting as a wetting agent could reduce surface tension, thereby decreasing contact angle. The reduced contact angle could increase the contact area, spreading and coverage of droplets on target surfaces, beneficial to pesticide deposition and retention^28^.

### Biological activity

Though avermectins have a broad pesticidal spectrum, avermectin B_2_ was reported to be more effective against root-knot nematode than avermectin B_1_^8^. The biological activities of formulations are influenced by many factors such as pest species and instar, formulation characteristic and experimental condition^31^. As shown in Table 2, the avermectin B_2_ EC had the highest toxicity against diamondback moths (*Plutella xylostella*) followed by solid nanodispersion. The toxicity of the solid nanodispersion was 2.6 and 12.8 times that of EW and WDG, respectively. A leaf-dip method was adopted in the test, so the results were influenced by the wetting and retention properties of formulations on the leaf surface. As proved by the bioassay results, the biological activity of the formulation on diamondback moths (*Plutella xylostella*) indeed had positive correlation with the formulation wettability, which was negatively correlated with surface tension and contact angle. The toxicity improvement of the avermectin B_2_ solid nanodispersion in foliar application mode is expected to expand its control on lepidopteran pests.

**Table 2:** Bioassay results of the avermectin B_2_ formulations to diamondback moths (*Plutella xylostella*). LC_50_: median lethal concentration.

Formulation	Toxicity regression equation	Correlation coefficient	LC_50_ (µg/mL)	95% confidence limit of LC_50_
Solid nanodispersion	Y = 0.619x + 4.5801	0.9719	4.77	2.62–8.66
EC	Y = 1.0478x + 4.5049	0.9753	2.97	1.90–4.64
EW	Y = 0.942x + 3.9734	0.9302	12.3	5.45–27.72
WDG	Y = 1.5851x + 2.1705	0.9846	60.96	35.14–105.78

As shown in Table 3, the toxicity of the solid nanodispersion to south root-knot nematode (*Meloidogyne incognita*) was 1.6, 1.7 and 4.2 times that of EC, EW and WDG, respectively. This bioassay result is not affected by the foliar wetting process. However, the small particle size as well as high dispersibility and uniformity was conducive to increasing the contact probability between pesticide and target organisms, thereby improving the biological activity of the formulation. In this investigation, the enhanced biological activity of the solid nanodispersion can be attributed to the small size and high surface-to-volume ratio of nanoparticles^32^. The application of nanoformulations with high bioavailability could substantially reduce pesticide dosage and cost, and further improve its environmental friendliness and economy.

**Table 3:** Bioassay results of the avermectin B_2_ formulations to south root-knot nematode (*Meloidogyne incognita*). LC_50_: median lethal concentration.

Formulation	Toxicity regression equation	Correlation coefficient	LC_50_ (µg/mL)	95% confidence limit of LC_50_
Solid nanodispersion	Y = 3.4553x + 7.7943	0.9707	0.1553	0.1375–0.1708
EC	Y = 7.6067x + 9.5914	0.9938	0.2491	0.2384–0.2597
EW	Y = 5.8829x + 8.4386	0.9824	0.2603	0.2482–0.2734
WDG	Y = 7.0338x + 6.2986	0.9750	0.6537	0.6267–0.6811

In this research, self-emulsifying as a simple and easy-to-scale-up technique was combined with solidification method to prepare the avermectin B_2_ solid nanodispersion. The solid nanodispersion is organic solvent-free and convenient for storage and transportation. The mean size and PDI of the avermectin B_2_ nanoparticles measured by DLS were 35.3 nm and 0.281, respectively. The surface tension and contact angles on hydrophobic surfaces of the solid nanodispersion were smaller than those of EW and WDG. In addition, the toxicities of the nanoformulation against diamondback moths and south root-knot nematode were more than 1.7 times that of EW and WDG. This highly effective and environmentally friendly solid nanodispersion has broad prospect in crop protection for improving pesticide efficacy and reducing residual pollution in agricultural products and environment. In addition, this investigation demonstrated that for foliage-applied pesticides, formulation bioavailability had positive correlation with wettability which was negatively correlated with surface tension and contact angle. The relationship between formulation parameters and biological activity can be used to guide the construction of novel pesticide formulations. It is of great significance to perfect the design theory of pesticide formulations.

## Materials and Methods

### Materials

Avermectin B_2_ technical material (TC, 95%, w/w), EC (5%, w/w), emulsion in water (EW, 2.5%, w/w) and water dispersible granule (WDG, 3.5%, w/w) were provided by Hebei Xingbai Agricultural Technology Co., Ltd. (Hebei, China). Sodium benzoate, α-lactose, glucose and sucrose were purchased from Sinopharm Chemical Reagent Co., Ltd. (Shanghai, China). Styryl phenol polyoxyethylene ether (emulsifier 600) and alkylphenol formaldehyde resin polyoxyethylene ether (emulsifier 700) were provided by Cangzhou Hongyuan Agrochemical Co., Ltd. (Hebei, China). Ethyl acetate, chromatographic grade methanol and acetonitrile were bought from J&K Scientific Ltd. (Beijing, China). Parafilm was purchased from Sigma-Aldrich Shanghai Trading Co., Ltd. (Shanghai, China). All the chemicals were used as received. Milli-Q water (18.2 MΩ.cm, TOC ≤ 4 ppb) was used in all analytical experiments.

### Preparation of the avermectin B_2_ solid nanodispersions

A self-emulsifying combined with carrier solidification technique was applied to prepare the avermectin B_2_ solid nanodispersions. Taking the 2.0% (w/w) avermectin B_2_ solid nanodispersion as an example, the detailed preparation procedure was as follows. Firstly, 0.47 g of avermectin B_2_, 0.94 g of emulsifier 600 and 0.94 g of emulsifier 700 were dissolved in 12 mL of ethyl acetate. Then the solution was added to 21.15 g of sodium benzoate. After stirring evenly, the mixture was dried in an oven (DHG-9070A, Shanghai Yiheng Science & Technology Co., Ltd., Shanghai, China) at 40 °C for 3 h to obtain the solid nanodispersion. During the optimization of formulation composition, the type of carrier and the amount of each component varied with the experimental design.

### Particle size and zeta potential measurements

The mean particle size, polydispersity index (PDI) and zeta potential of the samples were measured at room temperature using a Zetasizer Nano ZS 90 (Malvern, Worcestershire, UK). The sizes and PDIs were tested by dynamic light scattering (DLS). All the data were measured in triplicate and recorded as mean ± standard deviation (S.D.).

### pH measurement

The avermectin B_2_ solid nanodispersion was diluted into 0.01% (w/w) dispersion with pure water. Then a pH meter (HI2211, Hanna, Italy) was inserted into the dispersion and held until the data did not change. The average value of three measurements was recorded as mean ± standard deviation (S.D.).

### Morphological characterization of the particles

The morphologies of the avermectin B_2_ technical material and nanoparticles were visualized using a scanning electron microscope (SEM, JSM-7401F, JEOL, Tokyo, Japan). The aqueous dispersion of the sample was dropped on a freshly cleaned silicon slice. Then it was air-dried and coated with platinum by a sputter coater (ETD-800, Beijing Elaborate Technology Development Ltd., Beijing, China). The images were recorded in the low electron image (LEI) mode. In order to exclude the carrier interference, the sample area uncovered by carriers was selected to observe the morphology of the nanoparticles. The statistical particle size based on SEM image was calculated by a Nano Measurer software.

### Determination of the avermectin B_2_ content

The content of the active ingredient was analysed by high-performance liquid chromatography (HPLC, LC-20AD, Shimadzu, Kyoto, Japan) using a Ultimate XB-C_18_ column (5 um, 4.6 mm × 150 mm, Welch, Shanghai, China) at 30 °C. The mobile phase was composed of acetonitrile, methanol and water (40:40:20, v/v). The flow rate was 1.0 mL/min and the UV detector wavelength was 254 nm.

### Stability test

The storage stability of the solid nanodispersion was evaluated according to CIPAC MT 46 and GB/T 19136–2003. The freshly prepared solid nanodispersion powder was placed in bottles and sealed. Then the samples were stored in a refrigerator at 4 °C and an incubator (ZXDP-B2080, Shanghai Zhicheng Analytical Instrument Manufacturing Co., Ltd., Shanghai, China) at 54 °C, respectively. After 7-day storage at 4 °C and 14-day storage at 54 °C, the samples were taken out to measure their particle sizes and PDIs by DLS. The average value of three measurements was adopted.

### Surface tension measurement

The surface tensions of the formulations were measured using the du Nouy ring method with an automatic tensiometer (JK998BM, Zhongchen Digital Technic Apparatus Co., Ltd., Shanghai, China). Before each measurement, the platinum ring was cleaned by heating with an alcohol lamp. The ring was immersed in the 0.01% (w/w) avermectin B_2_ aqueous dispersions. After that, it was slowly lifted up. When the ring was about to leave the solution surface, liquid formed a film on the ring. The maximum force was recorded when the liquid film broke. The surface tension (γ) was calculated by the pre-programmed software in the tensiometer according to the following equation^33^.

$$\gamma =\frac{{\rm{P}}}{4\pi {\rm{R}}}{\rm{F}}$$

Where P is the force acting on the ring (N), R is the radius of the platinum ring (cm), F is the correction factor.

### Contact angle measurement

The surface microtopography of real leaves have a significant impact on the contact angle. In this research, both Parafilm and cabbage (*Brassica oleracea*) leaves were used as hydrophobic surfaces to evaluate the formulation wettability. The contact angles were measured by sessile drop method using a contact angle apparatus (JC2000D, Zhongchen Digital Technic Apparatus Co., Ltd., Shanghai, China) at room temperature. Seven microliters of the 0.01% (w/w) avermectin B_2_ aqueous dispersion was dropped onto the surface and balanced for five seconds. Then the contact angles were acquired by five point fitting method. The average value of four measurements was adopted.

### Bioassays

In the toxicity test on diamondback moth (*Plutella xylostella*), the leaf-dip method was adopted according to NY/T 1154.14–2008 and literatures^34^. The different avermectin B_2_ formulations were diluted with pure water to a series of concentrations (50 μg/mL, 20 μg/mL, 5 μg/mL, 2 μg/mL, 0.5 μg/mL) based on the content of active ingredient. Then cabbage (*Brassica oleracea*) leaves were immersed in the dispersions and pure water as a control for 10 s. After air-drying, the leaves were placed in a culture dish with a filter paper. Ten second-instar diamondback moth (*Plutella xylostella*) larvae were introduced into each dish, and three replications were carried out. In the experiment, the identification of second-instar larvae was based on their unique morphological characteristics according to GB/T 23392.3–2009: body length of 2.0–3.0 mm, head width of 0.244 mm, black head, body color from gray to canary yellow. The head is wider than body and the pronotum has two discontinuous U-shaped patterns. Mortality was assessed after treatment for 48 h. Larvae were considered dead if they could not be induced to move while touching with a brush. In the bioassay on south root-knot nematode (*Meloidogyne incognita*), second-stage juveniles hatched from the egg masses were used to perform the test. The avermectin B_2_ solid nanodispersion, EC and EW were diluted into 0.45 μg/mL, 0.4 μg/mL, 0.35 μg/mL, 0.3 μg/mL, 0.25 μg/mL, 0.2 μg/mL, 0.15 μg/mL and 0.1 μg/mL. The WDG formulation was dispersed into 0.9 μg/mL, 0.8 μg/mL, 0.7 μg/mL, 0.6 μg/mL, 0.5 μg/mL and 0.4 μg/mL. Then the pesticide aqueous dispersions were transferred to wells of 24-well tissue culture plates, and water containing 50 second-stage juveniles was added to each well. Each treatment was replicated four times with pure water as a control. Immotile and motile nematodes were counted after 24 h. Nematodes were considered immotile if they did not move while prodding with a fine needle. Concentration-mortality data were analysed using DPS 8.1 (Refine Information Technology Co., Ltd., Hangzhou, China). In both bioassay experiments, the median lethal concentrations (LC_50_) were derived from the toxicity regression equations.

### Statistical analysis

Data were analysed by one-way analysis of variance (ANOVA) and Duncan’s multiple range test. Statistical analysis was performed with the software package SPSS and a probability less than 0.05 was deemed statistically significant.

## Data Availability

All data generated or analyzed during this study are included in this article.
